# Cytotoxicity of Algae Extracts on Normal and Malignant Cells

**DOI:** 10.4061/2011/373519

**Published:** 2011-01-05

**Authors:** Jeremy Bechelli, Myra Coppage, Karen Rosell, Jane Liesveld

**Affiliations:** ^1^Department of Medicine and the James P. Wilmot Cancer Center, University of Rochester Medical Center, Rochester, NY 14642, USA; ^2^Department of Pathology and Laboratory Medicine, University of Rochester Medical Center, Rochester, NY 14642, USA

## Abstract

Algae preparations are commonly used in alternative medicine. We examined the effects of algae extracts on normal hematopoietic cells and leukemia cells. Ethanol extracts were prepared of *Dunaliella salina* (Dun), Astaxanthin (Ast), *Spirulina platensis* (Spir), and *Aphanizomenon flos-aquae* (AFA). Cell viability effects were completed by Annexin staining. Ast and AFA inhibited HL-60 and MV-4-11 whereas Dun and Spir had no effect. Primary AML blasts demonstrated increased apoptosis in AFA. Primary CLL cells showed apoptosis at 24 hours after exposure to Dun, Ast, Spir, and AFA. High AFA concentrations decreased viability of normal marrow cells. Normal CD34+ viability was inhibited by Dun. Dun and AFA inhibited BFU-E, but all extracts inhibited CFU-GM. Cell-cycle analysis of AML cell lines showed G0/G1 arrest in the presence of AFA. These data suggest that algae extracts may inhibit AML cell lines and leukemia blasts, but they may also have potential inhibitory effects on normal hematopoiesis.

## 1. Introduction

Plants provide a valuable source of therapeutic agents and bases for synthetic drugs. Several plant species have been studied for anticancer properties, and it is estimated that approximately 40%–50% of the drugs on the market today are either derived from natural products or are natural products themselves [1]. Nonetheless, comprehensive and systematic evaluation of “natural” products is required to demonstrate efficacy and safety for clinical use.

The Center for Complementary Health Studies defines complementary and alternative medicine (CAM) as “any health-improving technique outside of the mainstream of conventional medicine” [2]. The general public is very accepting of CAM, with recent reports stating that 34%–38% of the US adult population was using CAM [3], and 7.4% of the population had seen a CAM practitioner during the last 12 months [4]. 

There is growing interest among scientists to understand and potentially utilize traditional complementary medicinal compounds. Hundreds of plant products are available as nutritional supplements, many of which have not been scientifically evaluated. Several species of algae have received recent attention with claims of boosting the immune system and preventing cancer, but experimental evidence supporting such claims is limited. 

Based on the common use of algae or algae components in supplements to boost immunity and to affect oxidation status, our laboratory undertook a study of extracts and byproducts of four species of algae commonly used as nutritional supplements. Extracts of Spirulina (*Arthrospira platensis*) (Spir), *Dunaliella salina *(Dun), and *Aphanizomenon flos-aquae* (AFA) and an extract of Astaxanthin (Ast), a carotenoid derivative of the algae *Haematococcus pluvialis*, were evaluated using an *in vitro* assay system. Cancer cell lines, primary cancer cells, and normal cells were exposed to the compounds in culture medium and effects on proliferation, cell cycle, induction of reactive oxygen species (ROS), and apoptosis were examined. Effects of the compounds on hematopoietic progenitors were also tested in colony-forming assays for granulocyte-macrophage colony forming units (CFU-GM) and erythroid burst forming units, (BFU-E). Taken together, the data derived from these assays suggest that extracts from algae utilized as food supplements have the ability to inhibit AML cell lines and primary leukemia blasts but also demonstrate inhibitory effects on normal hematopoietic cells. Each algae extract demonstrated a different pattern of inhibition, and whether these extracts are inhibitory at a stem-cell level or would have differential effects on normal or leukemic cell growth in vivo remains to be determined.

## 2. Materials and Methods

### 2.1. Preparation of Algal Extracts and Cell Culture

Ast, Dun, and Spir were obtained from Valensa (Eustis, FL, USA), and AFA was obtained from e3live (Klamath Falls, OR, USA) and stored at 4°C. One gram of algae was added to 10 ml of 70% ethanol and incubated at 4°C on a shaker for 24 hours. The slurry was centrifuged at 400 g for 10 min at 4°C, and the supernatant was filtered through 413-grade filter paper. The resulting extract was stored in the dark at −80°C until used for experiments. All algae samples used throughout the study were consistent in regard to lot number; however, fresh extracts were prepared as needed. The concentration of extracts added to cells is represented in volume/volume (*μ*l/ml) measurements, because the actual concentration of bioactive constituents is currently unknown. 

Cell lines were purchased from the ATCC (Rockville, MD). Blood or marrow aspirates from patients with acute myelogenous leukemia (AML) with high blast percentages and chronic lymphocytic leukemia (CLL) with high white cell counts were obtained with informed consent as part of diagnostic procedures. Light-density cells were enriched by Ficoll-Hypaque centrifugation. Marrow aspirates were obtained from normal donors in accord with policies of the Research Subjects Review Board of the University of Rochester. Marrow cells were subjected to Ficoll-Hypaque density gradient centrifugation. The light-density layer was removed, washed, and resuspended in phosphate-buffered saline (PBS). CD34+ cells were isolated using Miltenyi Biotec MiniMACS magnetic bead cell separation columns (Auburn, CA, USA). Marrow cells (10^7^) in 300 ml PBS with EDTA/BSA were incubated with 100 *μ*l monoclonal microbead-conjugated anti-CD34 antibody (QBEND/10; Becton Dickinson) for 30 min at 4°C. Thereafter, the cells were washed and passed through a 30 *μ*m nylon mesh and separated in a column exposed to the magnetic field of the MACS device. The column was washed four times with sorting buffer (500 *μ*l aliquots) and removed from the separator. This process was then repeated, and the CD34+ cells and subsets were >95% pure as determined by FACS analysis. 

Leukemic cell lines, fresh AML samples, and light-density or CD34+ marrow or blood cells from normal donors were cultured in RPMI with 10% FBS.

### 2.2. Determination of Apoptosis

Annexin V staining was used as a measure of early apoptosis induction in both cell lines and primary samples. Annexin V staining kits were purchased from R&D Systems (Minneapolis, MN, USA). The staining protocol provided by the company was used. Briefly, Annexin V staining solution was prepared with use of 10X binding buffer (10%), annexin V-FITC (2%), and propidium iodide (10%) and incubated with 1 × 10^6^ cells for 15 min at room temperature in the dark. Cells were stored in binding buffer until subjected to fluorescence-activated cell-sorting analysis. Flow cytometry was performed on 10,000 cells with a FACSCalibur dual laser cytometer, and data analysis was performed using FloJo software.

### 2.3. CFU-GM and BFU-E Assay

Normal light-density bone marrow cells were treated with 8.0  *μ*l/ml or 15  *μ*l/ml of the algae extracts and incubated for 24 hours at 37°C. Cells were washed with PBS and 1 × 10^5^ viable cells were plated in 0.9% methylcellulose, 30% FBS, 2 mmol/l L-glutamine, 10^−4^ mol/L-mercaptoethanol, 1% BSA with 3 U/ml human erythropoietin, 10 ng/ml granulocyte-macrophage colony-stimulating factor (GM-CSF), 10 ng/ml interleukin 3 (IL-3), and 50 ng/ml SCF (stem cell factor or *c-kit* ligand). The methylcellulose mixture was purchased from Stem Cell Technologies (Vancouver, BC, Canada). Colonies were scored at Day 14 and were defined as >30 grouped cells.

### 2.4. Cell-Cycle Analysis and ROS Detection

Cell-cycle status was determined using propidium iodide staining. Cells were fixed in 80% ethanol on ice for 15 minutes and pelleted by centrifugation. Cells were stained with a solution of 50 ug/ml propidium iodide, 10 ug/ml RNase in 0.5 ml volume for 30 minutes at 37°C. Flow cytometry was performed as above. ROS was detected in AML blasts (12 × 10^6^) labeled with dihydrorhodamine 123 (Cayman Chemical, Ann Arbor, Michigan, USA) at 2.5 micromolar for 20 minutes at room temperature. Cells were washed and resuspended at 5 × 10^5^ per ml and plated 1 ml per well of a 24 well plate. To the wells, 15  *μ*l/ml of algae extract (Spir, Ast, or AFA) or ethanol (vehicle control) was added. Exposure to hydrogen peroxide was a positive control for oxidation. Plates were incubated for 2 hours, then wells were harvested and oxidation assessed by flow cytometry.

### 2.5. Statistical Considerations

Two-tailed paired *t*-tests were employed to determine the significance of differences between experimental conditions and control conditions. A *P*-value of <.05 was required for the determination of statistical significance. In all figures, *n* refers to the number of experiments performed using distinct samples.

## 3. Results

### 3.1. Effects of Algae Extracts on the Growth of Human Cancer Cell Lines

**Figure 1:**
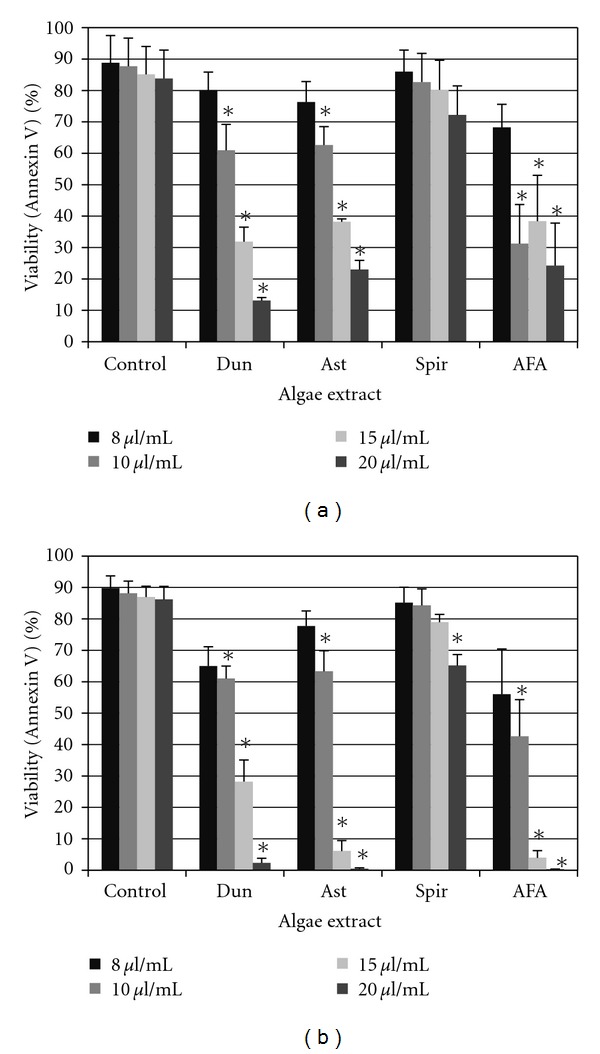
Effects of algae extracts on leukemia cell-line viability. HL60 cells (a) and MV-4-11 cells (b) were treated with algae extracts for 24 hours and analyzed by Annexin V assay for viability. Shown are mean values ± standard errors of three independent experiments (*indicates *P* < .05).

The binding of Annexin V to phosphatidylserine is a common assay used as a measure of drug toxicity as it reflects translocation from the inner to the outer leaflet of the plasma membrane, which is one of the earliest features of cellular death via apoptosis. The effects of extracts on the growth of the human leukemia cell line HL-60 and the biphenotypic B myelomonocytic leukemia cell line MV-4-11 are shown in Figure 1. Panels A and B demonstrate a dose-dependent decrease in viability in cells treated for 24 hours with algae extracts. Dun, Ast, and AFA inhibited growth of HL-60 cells (Figure 1(a)). Significant (*P* < .05) inhibition was observed at concentrations of Dun, Ast, and AFA extract starting at 10.0  *μ*l/ml. The HL-60 cells exposed to Spir extracts demonstrated a slight, but not significant, decrease in viability between treated and vehicle control groups. MV-4-11 cells (Figure 1(b)) were sensitive to exposure of Dun, Astaxanthin, and AFA as indicated by concentration-dependent reductions in viability starting with Dun at 8.0  *μ*l/mL and both AST and AFA at 10.0  *μ*l/ml (*P* < .05). The MV-4-11 cells exposed to Spir extracts demonstrated a significant decrease in viability between treated and vehicle control groups only at the highest doses tested, 20.0  *μ*l/ml. Viability data was confirmed using trypan blue staining (Data not shown).

### 3.2. Effects of Algae Extracts on the Viability of Primary Leukemia Cells

**Figure 2:**
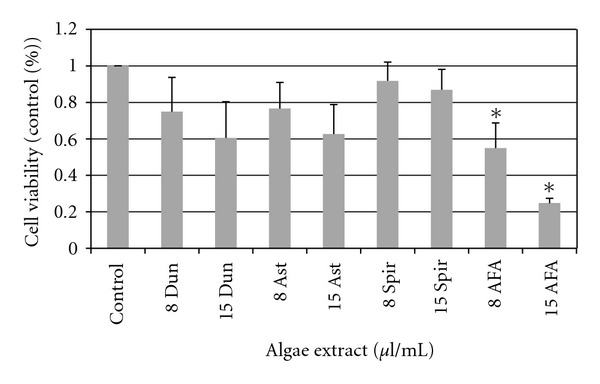
Viability of primary AML samples treated with algae extracts. Primary leukemic blasts were treated with 8  *μ*l/ml and 15  *μ*l/ml doses of algae extracts for 24 hours and assayed for viability via annexin V staining. Data shown is normalized to conditions with vehicle control added ± standard errors of three independent experiments (*indicates *P* < .05).

To examine potential antileukemic effects of algae extracts, primary AML blasts from 3 patients were cultured for 24 hours under increasing concentrations of algae extract and analyzed by flow cytometry for Annexin V (Figure 2). Primary AML blasts were sensitive to AFA starting at 15 *μ*l/ml in a dose-dependent manner; however, exposure to Spir, Dun, and Ast extracts did not induce apoptosis at concentrations up to 15 *μ*l/ml.

**Figure 3:**
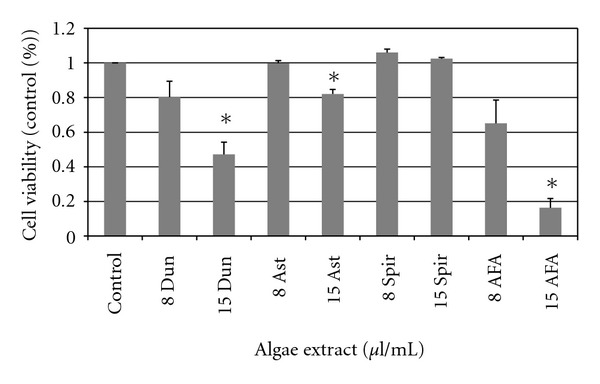
Viability of primary CLL samples treated with algae extracts. Primary leukemic blasts were treated with 8  *μ*l/ml and 15  *μ*l/ml doses of algae extracts for 24 hours and assayed for viability via annexin V staining. Data shown is normalized to conditions with vehicle control added ± standard errors of four independent experiments (*indicates *P* < .05).

Primary tumor cells from four CLL patients were tested using the same approach applied to the AML cells (Figure 3). Cell viability was significantly reduced upon exposure only to the highest dose (15 *μ*l/ml) of Dun, Ast, and AFA whereas Spir extract had no effect on the viability of primary CLL cells.

### 3.3. Effects of Algae Extracts on Viability of Normal LDBM and CD34+ Cells

**Figure 4:**
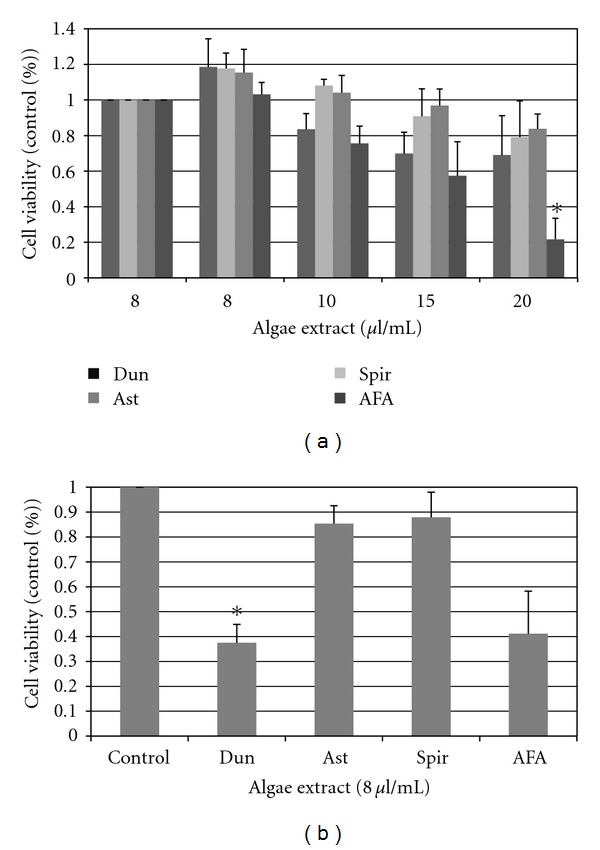
Viability of primary normal bone marrow cells and normal CD34+ cells treated with algae extracts. Primary light density mononuclear cells (a) were treated with 8.0 *μ*l/ml and 15 *μ*l/ml doses of algae extracts for 24 hours and assayed for viability via annexin V staining. Normal CD34+ cells (b) isolated from healthy volunteers were isolated and cultured for 24 hours in the presence of algae extracts and stained for anexin V. Data shown is normalized to conditions with vehicle control ± standard errors of three independent experiments (*indicates *P* < .05).

To examine potential death-inducing effects of algal extracts on normal light density bone marrow, cells from three healthy individuals were cultured in the presence of algae extracts for 24 hours and assayed using Annexin V and propidium iodide staining by flow cytometry (Figure 4(a)). AFA markedly decreased the viability of normal light density marrow cells at 20 *μ*l/ml. While not statistically significant, the other extracts were associated with only a slight decrease in viability.

To investigate the effects of algae extracts on normal CD34+ hematopoietic progenitor cells, we isolated CD34+ cells from the bone marrow of normal healthy volunteers and exposed these cells to 8.0 *μ*l/ml of extract for 24 hours *in vitro*. Ast and Spir extract resulted in no decrease in viability; however, Dun and AFA significantly increased the expression of phosphatidylserine as detected by Annexin V staining, indicating a decrease in cellular viability (Figure 4(b)). Interestingly, AFA did not significantly reduce the viability of hematopoietic cells in culture conditions (*P* = .075) as measured by Annexin V staining.

### 3.4. Effects of Algae Extracts on Normal Colony-Forming Unit Ability

**Figure 5:**
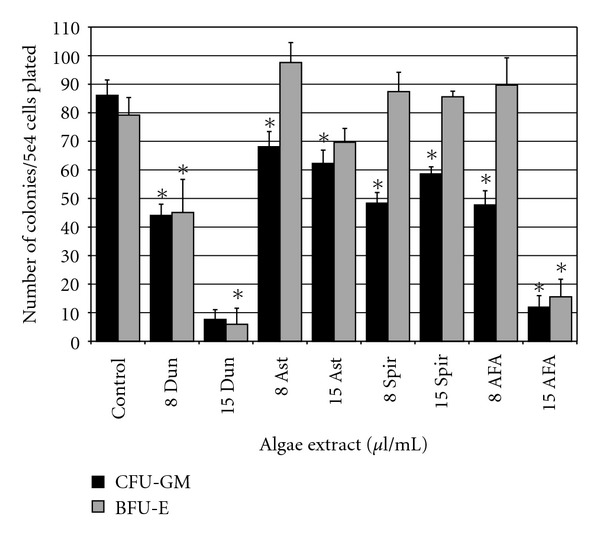
Effect of Algae extracts on day 14 CFU-GM and BFU-E from normal light-density bone marrow. The dose response of algae extracts on colony outgrowth when cultured with methylcellulose and 3 U/ml erythropoietin, 10 ng/ml GM-CSF, 10 ng/ml IL-3, 10 ng/ml IL-6, 10 ng/ml G-CSF, and 50 ng/ml stem-cell factor are shown. Data shown is normalized to conditions with vehicle control ± standard errors of three independent experiments (*indicates *P* < .05).

Clonogenic assays for granulocyte/macrophage and erythroid progenitor cells were used to evaluate toxicity of algae extracts for earlier progenitor cells. The growth of granulocyte-macrophage colony-forming units (CFU-GM) was inhibited by all extracts at both concentrations tested (Figure 5). Erythroid burst forming units (BFU-E) were inhibited by Dun at both tested concentrations and at the highest dose of AFA. Both CFU-GM and BFU-E were dramatically inhibited by the highest doses of Dun and AFA (*P* ≤ 5.0E^−6^).

### 3.5. Effects of Algae Extracts on Leukemic Cell-Cycle Arrest and ROS Induction

**Figure 6:**
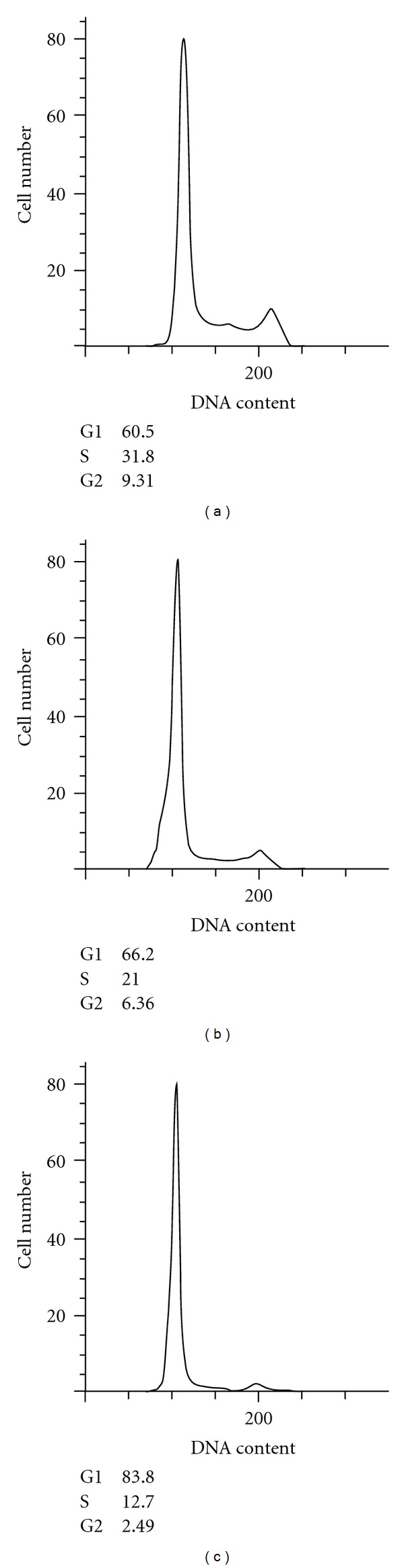
Cell-cycle distribution of HL-60 cells treated with AFA. HL60 cells were cultured with vehicle control (a), 8.0 *μ*l/ml AFA (b), and 15 *μ*l/ml AFA (c), for 24 hours, and DNA was stained with propidium iodide and analyzed by flow cytometry. Data shown is one representative experiment of HL60 cells treated with AFA for 24 hours.

**Figure 7:**
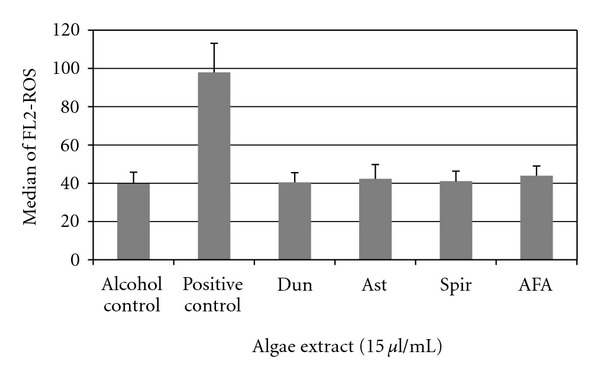
Peroxynitrite formation in AML blasts treated with algae extracts. Primary leukemic cells were cultured in the presence of 15 *μ*l/ml of the algae extract for two hours. Cells were labeled with dihydrorhodamine 123 for 20 minutes at room temperature and analyzed by flow cytometry (*indicates *P* < .05).

As a result of the observed inhibition of metabolic processes of leukemic cell lines, we tested whether algae extracts would alter or interrupt the cell cycle status of malignant cells. Cell cycle was assessed by DNA content analysis using flow cytometry in HL-60 leukemia cancer cells treated with AFA, Ast, Spir, or Dun. Figure 6 shows a representative cell-cycle distribution of HL-60 cells incubated in the absence or presence of AFA extract for 24 hours, the approximate doubling time of this cell line. In the absence of the extract, >40% of the cells were in either S or G2 phase. However, when treated with AFA (8.0 and 15 *μ*l/ml), a dose-dependent increase in the percentage of cells in G1 phase and concomitant decrease in S and G2/M were observed, supporting a G1 phase arrest. A similar increase of cells in G1 phase and were decrease in S and G2/M observed with Dun (8.0 and 15 *μ*l/ml) and 15 *μ*l/ml Ast (Data not shown). As expected, based on MTT results, Spirulina had no detectable effect on cell-cycle progression. We observed no increase in reactive oxygen species caused by exposure to algae extracts at any time point up to 2 hours. The hydrogen peroxide treated cells used as a positive control showed significant ROS formation using dihydrorhodamine 123 as an indicator of peroxynitrite formation (Figure 7).

## 4. Discussion

The two most common edible cyanobacteria include Spirulina and AFA [5], both of which contain phycocyanin, a molecule shown to induce apoptosis in the chronic myeloid leukemia cell line, K562 [6] and other types of cancer [7–9]. Spirulina has been taken as a nutritional supplement for many years and has shown no undesirable side effects [10]. It has been dubbed nature's richest and most complete source of nutrition [11]. Orally administered spirulina extract has been shown to enhance tumoricidal NK activation through the MyD88 pathway, and spirulina exerted a synergistic antitumor activity with BCG-cell wall skeleton when used as immunotherapy of melanoma [12]. Our studies exposing cells to Spir extract demonstrated little effect on the growth or viability of either cancer cell lines or normal hematopoietic or stem cells at the doses tested. The only significant effects observed were a decrease in MV-4-11 cell viability at high doses (20 *μ*l/ml) and an inhibition of CFU-GM.

Hart et al. showed that the extract of *A. flos-aquae *(AFA) is a potent *in vitro *activator of NK cells, which are capable of killing some tumor cells without prior sensitization to antigen. However, this effect appears to be dependent on accessory cells as activation was not observed on isolated NK cells [5]. Pugh and Pasco demonstrated that AFA extract activated the monocyte cell line THP-1 [13]. Increased levels of both IL-1*β* and TNF-*α* in cells exposed to 0.5 pg/ml of extract were detected. 

AFA is known to produce several toxins including the hepatotoxin cylindrospermopsin (CYN) [14], and paralytic shellfish poisons, a potent neurotoxin [15, 16]. In contrast to our findings with Spir, our studies show that AFA extract alone increased apoptosis in all of the cancer cell lines we tested. In addition, significant toxicity was observed for normal bone marrow cells as well as for erythroid and granulocyte-macrophage progenitors. 

Dunaliella (Dun) provides a plenteous source of natural carotenoids that are in high demand due to applications in nutrition and medicine by consumers today. Dunaliella carotene, extracted from Dunaliella alga, showed no mortality or treatment-related adverse clinical effects throughout a 90-day subchronic toxicity study performed in F344 rats [17]. Murthy et al. showed a beneficial effect of dunaliella carotenoid compared to synthetic carotene as an antioxidant through decreased antioxidant enzymes catalase, superoxide dismutase, and peroxidase compared to controls [18]. Our studies showed a significant effect on apoptosis (Annexin V staining) by exposure of leukemic cell lines or primary tumor cells to Dun extract. We also observed that higher doses of Dun were associated with decreased viability of CD34+ stem cells. In addition Dun exposure also induced a significant decrease in both CFU-GM and BFU-E colony formation.

The astaxanthin (AST) rich *H. pluvialis* extract has inhibited cell growth in a dose- and time-dependent manner, by arresting cell-cycle progression and by promoting apoptosis in the HCT-116 colon cancer line [19]. Apoptosis was upregulated by modifying the ratio of Bax/Bcl-2 and Bcl-XL, and the phosphorylation of p38, JNK, and ERK1/2 was increased [19]. Other carotenoids derived from algae induce cell-cycle arrest through alteration in cyclin D2, D2, CDK4, and CDK6, and they may alter the expression of Bcl-1, XIAP, and survivin. Caspase activation has also been reported [20]. Ishikawa et al. showed Ast had mild inhibitory effects on HTLV-1-infected T-cell lines, a model system for adult T-cell leukemia [20]. It has been suggested that astaxanthin may improve antitumor immune responses by inhibiting lipid peroxidation induced by stress. Astaxanthin has been shown to protect several cell types from oxidative damage including neuronal cells [21], cervical cancer cells [22], and human umbilical-vein endothelial cells [23–27]. The ultimate effect of algal compounds and their metabolites on oxidative status is often indeterminate. Induction of apopotosis via generation of reactive oxygen species in human leukemia cell lines has been demonstrated [28], but other enzymatic extracts from algae have been found to protect against DNA damage induced by H_2_O_2_ [29] or to demonstrate chemopreventive properties through inhibition of nitric oxide and other inflammatory mediators such as TNF-alpha and COX-2 [30]. To address cellular ROS in our model, we treated primary AML blasts with algal extracts using dihydrorhodamine 123 as an indicator of peroxynitrite formation. We did not observe a cellular increase in reactive oxygen species caused by exposure to any of the algae extracts at a two-hour time point, suggesting that apoptosis was not induced by this pathway of reactive oxygen generation (Figure 7). Similar results were obtained for the cell lines HL-60 and MV-4-11 (Data not shown). 

In our studies, astaxanthin (AST) exposure resulted in no decrease in viability of normal light density marrow cells, or of normal CD34+ cells and no inhibitory effect on BFU-E colony formation or primary AML blasts. However, significant reductions in viability were observed in the leukemic cell lines MV-4-11 and HL-60, as well as primary chronic lymphocytic leukemia cells. 

The primary algae samples used were consistent throughout this study; however, because the constituents are largely unknown, we expect considerable variability between batches and manufacturers within the health food industry. This lack of uniformity compounded with several unknown bioactive components is a significant concern for health care providers, where patients take various herbal or algal supplements. To address this aspect of herbal pharmocology, we have used algae readily available to the general public, sold by the health food industry. Furthermore, use of extracts does not allow for determination of components or secondary metabolites, which might be responsible for the effects seen. For example, secondary metabolites of various algae have been found to affect cell adhesion [31]. In other cases, polysaccharide or deacetylated components have been responsible for activities reported [20]. 

In the present study, we demonstrated that ethanol extracts from astaxanthin (AST), *Dunaliella salina* (Dun), and *aphanizomenon flos-aquae *(AFA) inhibit both HL-60 and MV4-11 leukemic cell lines. Using primary patient samples of AML and CLL, we identified doses of AFA that significantly reduced viability of malignant cells in both types of leukemia. Cells from chronic lymphocytic leukemia patients were sensitive to all of the extracts tested except Spirulina. High doses of Dun, Ast, and AFA significantly reduced the viability of CLL cells in culture. 

While some cyano- and carotenoid-algae strongly inhibit the AML cell lines HL-60 and MV-4-11 as well as several primary leukemia blasts, we also observed cytotoxic effects on normal hematopoietic cells as demonstrated by Annexin V staining of light density peripheral blood cells. Of the four extracts we examined, each showed a pattern of inhibition that was different, indicating there are probably several compounds and mechanisms that are active against the malignant cells as well as normal light density marrow cells and hematopoietic progenitor cells. The role, if any, that these extracts have on normal or leukemic stem cells *in vivo* remains to be determined.

It is recognized that these *in vitro* studies of direct cytotoxicity are limited in that they are conducted apart from the tumor microenvironment and immunologic milieu of the host. Furthermore, these algal compounds are ingested in forms that do not allow measurement of feasible serum or tissue concentrations to compare with the *in vitro* concentrations employed here.

## 5. Conclusion

Taken together, these data suggest that extracts from algae have the potential to inhibit AML cell lines and primary leukemia blasts but also to inhibit normal hematopoietic cells. Each algae extract demonstrated a different pattern of inhibition, and whether these extracts are inhibitory at a stem-cell level or would have differential effects on normal or leukemic cell growth in vivo remains to be determined.
